# The Sequencing Quality Control 2 study: establishing community standards for sequencing in precision medicine

**DOI:** 10.1186/s13059-021-02528-3

**Published:** 2021-11-08

**Authors:** Tim R. Mercer, Joshua Xu, Christopher E. Mason, Weida Tong, on behalf of the MAQC/SEQC2 Consortium

**Affiliations:** 1 Australian Institute of Bioengineering and Nanotechnology, University of Queensland, Brisbane, Australia; 2 Genomics and Epigenetics Division, Garvan Institute of Medical Research, Sydney, NSW Australia; 3 Division of Bioinformatics and Biostatistics, National Center for Toxicological Research, US Food and Drug Administration, Jefferson, AR USA; 4 Department of Physiology and Biophysics, Weill Cornell Medicine, New York, NY USA; 5 The HRH Prince Alwaleed Bin Talal Bin Abdulaziz Alsaud Institute for Computational Biomedicine, Weill Cornell Medicine, New York, NY USA; 6 The WorldQuant Initiative for Quantitative Prediction, Weill Cornell Medicine, New York, NY USA

The MicroArray and Sequencing Quality Control (MAQC) consortium is a FDA-led, community-wide effort to evaluate the use of genomic technologies in clinical applications [1]. This evaluation includes the benchmarking of NGS technologies, the development of reference materials, and understanding the experimental and bioinformatic variables that impact the accuracy and reproducibility of large genomic datasets. These outcomes are ultimately used to inform best-practice guidelines, regulatory considerations, and foster further improvements in genomic technologies and computational methods [2].

The MAQC consortium has been ongoing for almost 16 years with four projects (MAQC I-IV). The founding project, MAQC Phase I, was initiated in 2005 by the FDA’s National Center for Toxicological Research (NCTR) to evaluate the reliability of microarray technologies that were being increasingly used in research, clinical diagnosis, and drug development and thus posed an urgency for the FDA to address the regulatory implication of the technology [3, 4]. In 2010, the MAQC consortium launched the SEQC (Sequencing Quality Control, known as MAQC III) project to investigate emerging next-generation sequencing (NGS) technologies. This SEQC project established best-practice use of RNA sequencing (RNA-seq) for measuring gene expression, compared RNA-seq performance to microarrays [5], evaluated the inter-platform reproducibility of NGS technologies [6], and evaluated the bioinformatic tools increasingly required to analyze large and complex RNA-seq data-sets [7].

## The Sequencing Quality Control Phase 2 (SEQC2) consortium

Most recently, the MAQC consortium completed its fourth and largest research project, known as SEQC2 (Sequencing Quality Control Phase 2; 2016-2021), which encompassed more than 300 participating scientists from 150 industry, academic, and government organizations across the world.

The SEQC2 project had three specific aims: (i) develop reference materials that could be shared by laboratories for standardized evaluation of NGS technologies, (ii) benchmark the impact of experimental and bioinformatic variables on the generation and analysis of NGS data and, (iii) evaluate inter- and intra-lab reproducibility of NGS technologies across different laboratories [8].

**Fig. 1:**
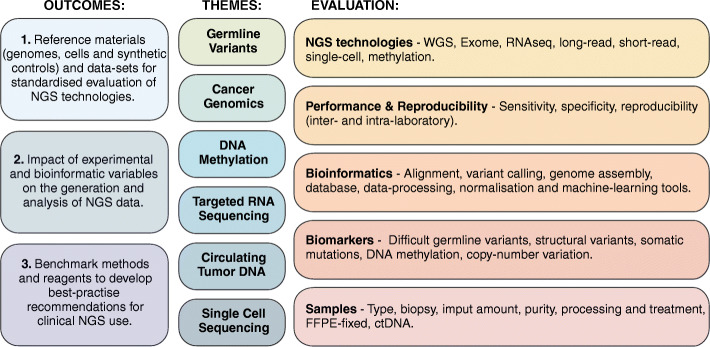
Schematic overview of the MAQC/SEQC2 project organization, aims and methods used for analytical validation of NGS technologies

The SEQC2 project is organized into six themes, each focusing on a different clinical application, including genome sequencing, cancer genomics, single-cell sequencing, circulating tumor DNA, epigenetics (eDNA methylation), and targeted RNA sequencing (see Fig. 1). Together, the diverse research and clinical laboratories that participated in the SEQC2 evaluated the performance of these differing NGS applications and built consensus standards for their best-practice use in clinical settings.

## Germline variants

Reproducible germline variant detection with whole genome sequencing (WGS) is vital for the implementation of precision medicine. However, the detection of variants in repetitive and difficult regions of the genome remains challenging, despite these regions harboring known, disease-associated genes with clinical importance. The WGS workflow is also lengthy and complex, with each step, from sample preparation, sequencing, and bioinformatic analysis affecting the diagnosis of germline variants.

To evaluate the detection of germline variants, SEQC2 performed WGS on reference genomes from two human populations using most major platforms and methods, including PCR-free, short-read, long-read, whole-genome, and targeted exome sequencing methods. Variants were then detected from the resulting sequencing data using more than fifty combinations of alignment and variant-calling bioinformatic tools. Performance was evaluated according to read alignment and coverage, error rates, and the sensitivity and specificity for correctly detecting known germline and structural variants in the reference genomes. These metrics were then stratified across genome, regions including repeats, transposons, duplicated, and challenging regions of the human genome [8].

The analysis found that the bioinformatic workflow, including alignment and variant-calling tools, had the largest impact on reproducibility between laboratories. For example, most errors were false negatives that were missed by variant callers. The detection of insertions and deletions (indels) was particularly challenging, and larger, complex structural variants were routinely missed by variant callers. This highlights the primary sources of variability in the detection of germline variants and the need for improved and standardized bioinformatics workflows to support the use of WGS in precision medicine.

These studies showed the reliable detection of variants in difficult, repetitive, or polymorphic human genome regions remains challenging. Given that natural genomes are unable to provide a clear reference standard for these difficult regions, SEQC2 developed synthetic controls that provide an unambiguous representation of difficult sequences, including complex variants, viral and transposon insertions, duplications, translocations, haplotype blocks, and immune receptors. These synthetic controls were used to benchmark the performance of diverse sequencing technologies in resolving these difficult regions and provide best-practice guidelines to optimize analysis that ultimately expands diagnostic yield of WGS into these difficult regions.

## Cancer genomics

NGS is being increasingly used in precision oncology, where the diagnosis of cancer mutations informs patient prognosis and treatment. However, the detection of somatic mutations can be difficult due to their low-frequency and the impact of pre-analytical variables, such as biopsy type, purity, and input amount. To evaluate the detection of somatic mutations, the SEQC2 conduct two comprehensive analyses, one was focused on WGS which is emerging as a comprehensive technology in cancer genomics and the other was on oncopanel sequencing which is the default application practiced in many clinical settings. The former was relied on a paired tumor and normal tissues as a reference sample while the latter assembled mock tumor and normal reference samples by mixing cell-line DNA samples at different ratios to emulate different mutation frequencies [9].

Targeted gene panels can improve the sensitivity for detecting somatic mutations by focusing sequencing coverage on genes that are causatively associated with cancer. However, panel design can limit performance, and the additional enrichment step can introduce bias. The SEQC2 benchmarked eight major commercial gene panels to inform best practice guidelines for targeted sequencing in precision oncology, as well as evaluated the measurement of tumor mutational burden to encourage harmonization across test platforms [10].

Clinical laboratories routinely use formalin-fixed paraffin-embedded (FFPE) methods to preserve solid tumor biopsy samples. However, FFPE preparation can cause the damage and fragmentation of DNA fragments that confounds the diagnosis of somatic mutations. To evaluate the impact of this pre-analytical variable, a subset of reference materials were prepared at differing durations of FFPE-fixation [11]. Targeted sequencing of these FFPE reference materials identified the profile of false-positive errors resulting from fixation damage and showed damage was greatest at block surfaces and at increased with the duration of FFPE fixation.

The cancer genome often harbors large and complex mutations that result from genome instability. WGS can be required to diagnose these complex mutations, including translocations, loss-of-heterozygosity, and gene amplifications and deletions [12]. The SEQC2 consortium performed WGS using the reference tumor samples to understand the variables that impact diagnoses. Although the WGS workflow could be divided into different steps (such as sample and library preparation, sequencing and bioinformatic analysis), the study found that each step is highly integrated and interdependent, and clinical validation is necessary across the entire sample-to-result workflow [13].

## Circulating tumor DNA assays

Cancers can release DNA fragments (ctDNA) into the circulatory system that indicate tumor tissue origin, size, and stage. CtDNA sequencing provides a non-invasive sample for diagnosing cancer, monitoring treatment efficacy, and possible recurrence. However, the detection of rare somatic mutations from limited ctDNA input amounts is difficult, and PCR biases, poor alignment, and sequencing errors can confound accurate analysis.

To understand the variables that impact ctDNA sequencing, SEQC2 tested the performance of five leading ctDNA assays across twelve participating laboratories [14]. This proficiency study used contrived human ctDNA reference materials to model sensitivity and the impact of variables [15]. Notably, the study found that diagnosis of ctDNA somatic mutations at frequencies lower than 0.5% became increasingly unreliable and difficult across all assays. Further analysis of simulated and synthetic control DNA experiments suggests this limit was imposed by low ctDNA input amounts and could not be improved by further sequencing, but instead, new technical innovations are required to improve sensitivity.

## Targeted RNA sequencing

Due to the size and complexity of the transcriptome, rare or complex genes are often poorly detected using RNA sequencing resulting in poor sensitivity. However, targeted RNA-sequencing enriches for genes of interest prior to sequencing, thereby achieving increased sequencing coverage that can sensitively detect genes of interest and resolve complex spliced isoforms. Given these advantages, targeted RNA sequencing is being increasingly adopted to profile gene expression and diagnose fusion genes in cancer.

To evaluate the impact of variables during the targeted RNAseq workflow, SEQC2 analyzed different panel designs and protocols using RNA harvested from reference cell line mixtures across six participating laboratories. This evaluation included a comparison of long- and short-read sequencing methods and their relative ability to resolve novel fusion genes that result from complex chromosomal rearrangements. However, while targeted RNAseq protocols show high sensitivity and performance, the complexity of transcripts can result in poor specificity, with many false-positive spliced isoforms and novel fusion genes detected. This suggests that while targeted RNA sequencing can reliably diagnose known fusion genes, the diagnosis of novel or complex fusion and spliced genes remains challenging.

## DNA methylation

Epigenetic modifications, such as DNA methylation, have key roles in chromatin dynamics and the regulation of gene expression. The detection of DNA modifications can indicate cellular identity, development, and progression of various diseases and is being increasingly used for the diagnosis of cancer. However, the measurement of DNA methylation using alternative methods has differing advantages and limitations, and an evaluation is needed to standardize genome-wide methylation sequencing applications in clinical research.

The SEQC2 evaluated the genome-wide methylation profile of reference cell lines using a range of common bisulfite and oxidative-bisulfite sequencing methods, as well as new enzymatic methods for de-amination of cytosines (such as EM-Seq). The study also evaluated the use of nanopore sequencing methods that can directly detect modified bases, as well as the use of ATAC-seq protocols to profile chromatin accessibility. These NGS-based methods were further compared to established DNA methylation microarray assays within and between laboratories [16].

This SEQC2 proficiency study across six laboratories provided the first analytical comparison of these differing protocols and informed best-practice recommendations for clinical cytosine methylation and hydroxyl-methylation studies. In addition, the generation of reference datasets within the study provides a useful resource to benchmark and optimize bioinformatic workflows for detecting DNA methylation.

## Single-cell sequencing

Single-cell sequencing methods can measure gene expression and chromatin accessibility within individual cells. At this resolution, rare cell populations can be identified, and the cellular heterogeneity that drives cancer evolution and drug resistance can be measured. However, a diverse range of single-cell sequencing platforms and bioinformatic tools have been published in recent years, each with distinct capabilities, bias, and costs. Understanding the impact of these variables in single-cell sequencing is needed to integrate large cell atlases and achieve sufficient standardization of single-cell technologies for clinical applications.

The SEQC2 undertook a multi-center proficiency study to evaluate a wide range of single-cell protocols and bioinformatic tools using mixtures of reference cell lines [17]. Comparisons between protocols showed marked differences in RNA capture efficiency, library complexity, and the final measurement of gene expression. Bioinformatic tools also markedly impacted performance, with batch-effect correction biasing the ability to resolve populations and detect cell-markers. Nevertheless, reproducibility across laboratories was high when using standardized workflows and support the clinical translation of single-cell sequencing technologies.

## Conclusions

The main SEQC2 outcomes are reference materials and reference datasets which can be applied to evaluate a broad range of NGS technologies of today and tomorrow to establish best practice and support regulatory framework development. NGS is being increasingly adopted for the clinical diagnosis of disease and drag development, and it is critical for the research and clinical community to understand sensitivity, accuracy, and reproducibility of NGS in routine application. Over the past 10 years, the SEQC and SEQC2 projects undertaken by the MAQC consortium have performed analytical validation of NGS technologies across a diverse international network of research and clinical laboratories to support this real-world adoption.

The ambition of the SEQC2 project is to support the translation of emerging NGS technologies into routine clinical practice. This includes the analyses of pre-analytical variables, such as sample type, preparation, and input amount, as well as post-analytical variables, such as the impact of different bioinformatic tools on the interpretation of complex NGS datasets. These pre- and post-analytical variables are often overlooked during proof-of-principle demonstrations by test developers, but markedly impact test performance.

The SEQC2 project has also established reference materials that are commercially available as an ongoing resource for the research and clinical community. These materials enable scientists to establish and benchmark their NGS workflow, compare performance with consortium data [10, 15], and guide efforts by related scientific communities, such as the Association of Biomolecular Resource Facilities (ABRF). Similarly, the large number of datasets and protocols generated during SEQC2 are available as an accessible resource for ongoing development of bioinformatic tools [12]. However, despite these resources, final clinical validation of NGS assays using patient samples is required prior to clinical use.

The SEQC2 project has highlighted the variables that impact the accuracy and reliability of NGS across a range of applications. We anticipate these findings will inform the interpretation and analysis of genome data in regulatory practice. Previous findings from MAQC have been incorporated into draft FDA guidance for pharmacogenomics and in vitro diagnostics, as well as the use of genetic variant databases to support germline disease diagnosis [18, 19]. This has contributed to a regulatory understanding of genomic data that is now routinely submitted as part of medical product applications, with drug approvals increasingly incorporating genotypes in indications on product labels.

More broadly, the success of the SEQC2 also reflects the continued efforts of an enduring international collaboration of scientists from different backgrounds in academia, industry, and government that together form the MAQC consortium. The project has proven a template for community-wide and open-science efforts seeking to understand the performance of NGS technologies across diverse clinical and research laboratory contexts. Together these scientists aim to support the translation of rapidly evolving NGS technologies that will ultimately increase our understanding of disease, improve the diagnosis and care of patients, and benefit human health.
